# Sky-High Thyroglobulin Level Following Thyroid Lobectomy Without Evidence of Metastatic Disease

**DOI:** 10.1016/j.aed.2025.09.017

**Published:** 2025-09-30

**Authors:** Ibrahim Ajwah, Wael Alzahrani, Heather Lochnan

**Affiliations:** 1 Department of Internal Medicine, Division of Endocrinology, King Salman Armed Forces Hospital, Tabuk, Saudi Arabia; 2 Division of Endocrinology, Department of Internal Medicine, Riyadh, Saudi Arabia; 3 Division of Endocrinology and Metabolism, Department of Medicine, The Ottawa Hospital, Ottawa, Canada; 4 Faculty of Medicine, University of Ottawa, Ottawa, Canada

**Keywords:** thyroid nodule, thyroid cancer, thyroid lobectomy, thyroglobulin

## Abstract

**Background/Objective:**

Thyroglobulin (Tg) serves as a crucial indicator for monitoring recurrence in patients with differentiated thyroid cancer following total thyroidectomy and radioactive iodine therapy. The utility of following Tg after thyroid lobectomy (TL) is debatable. There appears to be insufficient evidence to establish a specific Tg cutoff that can reliably detect persistent or recurrent disease after TL. The objective of this report is to describe a patient with an unexpectedly elevated Tg level following thyroid lobectomy for low-risk papillary thyroid carcinoma.

**Case Report:**

A 41-year-old female patient underwent a left thyroid lobectomy for low-risk classic papillary thyroid carcinoma. Her postoperative assessment reveals an unexpectedly elevated Tg at 4055 ng/mL (normal range: 3.5-77 ng/mL) with a negative Tg antibody <22 IU/mL (kIU/L). This is confirmed through repeated tests using multiple techniques to rule out laboratory errors and interference. Metastatic workup, including thyroid sonographic assessment and biopsy, chest computed tomography, and an 18F-fluorodeoxyglucose positron emission tomography scan, yielded negative results. Following thyroidectomy, the Tg level decreases dramatically to 0.7 ng/mL (0.7 μg/L).

**Discussion:**

Significantly elevated Tg levels after TL warrant careful consideration, but it is crucial to exclude potential laboratory errors and assay interference; additionally, it is essential to rule out underlying metastatic disease.

**Conclusion:**

Measuring Tg and Tg antibody levels post-TL can provide a baseline for future reference. However, this case illustrates that high levels of Tg can be seen in the absence of thyroid cancer and therefore cannot reliably predict the risk of recurrence.

**Highlights**

• A significantly elevated thyroglobulin (Tg) level after thyroid lobectomy is a rare finding that requires careful evaluation
• It is essential to eliminate potential laboratory errors by repeating the serum Tg test and checking for interference from TgABs or heterophilic antibodies
• Although it is rare for low-risk differentiated thyroid cancer to metastasize, it is essential to rule out underlying metastatic disease when there is such an unusual sky-high Tg level

**Clinical Relevance**

This case highlights challenges in interpreting Tg levels postlobectomy. Clinicians should consider laboratory errors and nonmetastatic causes of elevated Tg and avoid aggressive interventions based solely on Tg levels, emphasizing individualized management.

## Introduction

Thyroid cancer is the most common endocrine cancer, and there has been a global increase in its incidence, which has grown by 313% over the past 4 decades. In 2023, approximately 43 720 new cases of thyroid carcinoma are anticipated.^1^

Despite this rise in incidence, mortality rates for thyroid cancer have remained low, at 0.5 per 100 000 U.S. persons per year.^2^

Since the American Thyroid Association (ATA) released its 2015 guidelines for managing differentiated thyroid cancer (DTC), there has been a significant shift in DTC management. The use of thyroid lobectomy (TL) has become more prevalent. According to the ATA guidelines, TL is recommended as the preferred treatment for very-low-risk DTC smaller than 1 cm and is considered an equivalent option to total thyroidectomy (TT) for larger low-risk DTC measuring 1 to 4 cm.^3^

With the changes in guideline recommendations and the introduction of new concepts, such as a risk-adapted approach and the idea that "less is more" in managing patients with DTC, the utility of TL has increased. However, several challenges also arise postoperatively, such as interpreting the post-TL thyroglobulin (Tg) level.^4^

In this report, we present a patient with low-risk papillary thyroid carcinoma (PTC) who was treated with TL and found to have an unexpectedly very high Tg level without laboratory interference in the absence of any structural disease, which reduced to the expected range after completion of thyroidectomy.

## Case Presentation

**Fig. 1:**
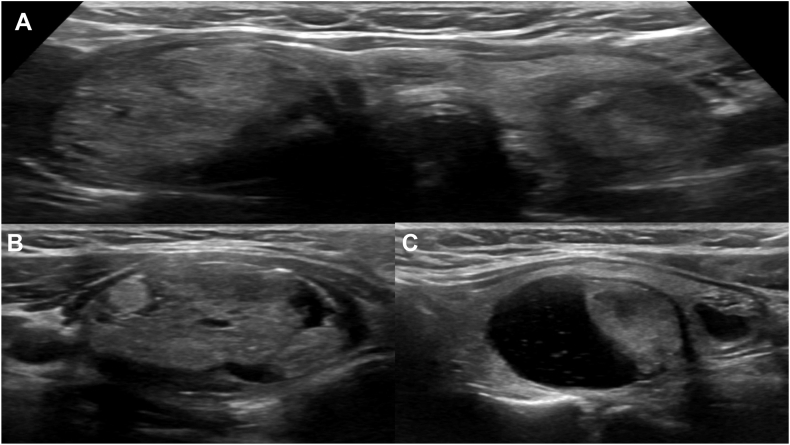
Preoperative thyroid ultrasound. *A*, The transverse view of the thyroid ultrasound shows bilateral thyroid nodules. *B*, *Right* thyroid nodule (32 mm)—isoechoic, mixed cystic and solid, wider than tall, with a smooth margin and punctate echogenic foci (TIRADS 4). *C*, *Left* thyroid nodule (27 mm)—mixed cystic and solid, hypoechoic, taller than wide, with a smooth margin, and without echogenic foci (TIRADS 5). *TIRADS* = Thyroid Imaging Reporting and Data System.

We present a female in her 40s with a medical history of IgA nephropathy and thyroid nodules, noted several years ago without any compressive symptoms, who reported no dysphagia, odynophagia, voice changes, or weight loss. There was no history of significant radiation exposure or family history of thyroid cancer. Two years earlier, she observed new changes in her neck. A sonography assessment using the Thyroid Imaging Reporting and Data System (TI-RADS) revealed a single nodule in the right thyroid lobe (R1), measuring 3.2 cm and classified as TI-RADS 4, along with 3 nodules in the left lobe (L1-L3); the largest (L1) measured 2.7 cm and was classified as TI-RADS 5 (Fig. 1 *A*–*C*). Fine needle aspiration (FNA) cytology results indicated a benign right thyroid nodule (Bethesda category II), while L1 was suspicious for PTC (Bethesda category V), L2 had nondiagnostic cytology results (Bethesda category I), and L3 was classified as benign (Bethesda category II). Upon review of the biopsy results and explaining the available treatment options, she was scheduled for left TL. Following the operation, she was referred to the endocrine clinic for follow-up surveillance.

On physical examination, she presented with a healed thyroidectomy scar and an asymmetric thyroid gland, notable for right lobe enlargement. The gland was soft and nontender, with no appreciable lymphadenopathy. Her surgical pathology was reviewed to assess her initial risk stratification per the latest ATA 2015 guidelines. The findings revealed a unifocal PTC measuring 2.7 cm. It was noted that no high-risk features were present, such as extrathyroidal extension or lymphovascular invasion, and the surgical margins were free of malignant cells. Her postoperative biochemical assessment showed a serum thyrotropin (TSH) of 3.8 μIU/mL (3.8 mIU/L) (normal range: 0.27-4.20 μIU/mL; 0.27-4.20 mIU/L), and the Tg level was dramatically elevated to 4055 ng/mL (4055 μg/L) (normal range: 3.5-77 ng/mL; 3.5-77 μg/L) with a negative Tg antibody <22 IU/mL (kIU/L). A neck ultrasound (US) was unremarkable. The original tumor staging was American Joint Committee on Cancer eighth edition stage I, and the ATA risk stratification was low risk with a biochemically incomplete response to therapy.

Her overall risk remains low, and the results of the preoperative thyroid US and FNA cytology of the right thyroid nodule are reassuring. Additionally, her postoperative TSH level is within the normal reference range. Therefore, we decided not to initiate TSH suppression therapy or plan any further surgical interventions and continued to be monitored clinically, biochemically with serial Tg/TgABs, and structurally with thyroid US.

**Fig. 2:**
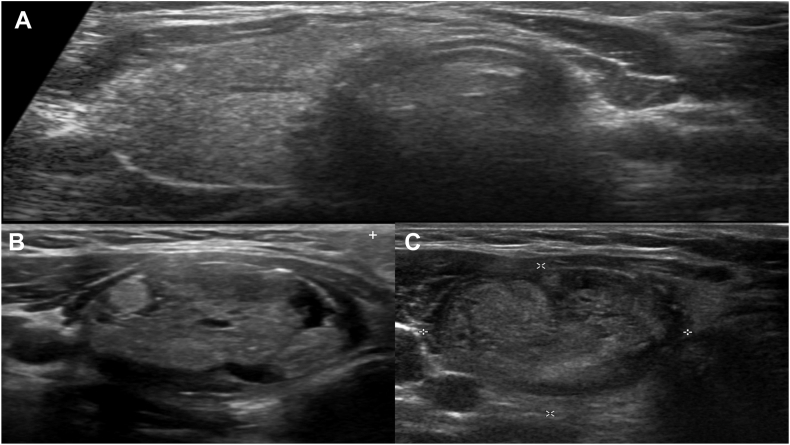
Postoperative thyroid ultrasound. *A*, The transverse view of the thyroid ultrasound shows a clear *left* thyroid bed following a *left* thyroid lobectomy. *B*, Baseline *right* thyroid nodule (32 mm)—isoechoic, mixed cystic and solid, wider than tall, with a smooth margin and punctate echogenic foci (TIRADS-4). *C*, Two-year follow-up of the *right* thyroid nodule (32 mm)—hypoechoic, solid, wider than tall, with a smooth margin and punctate echogenic foci and echogenic foci (TIRADS-5). *TIRADS* = Thyroid Imaging Reporting and Data System.

**Fig. 3:**
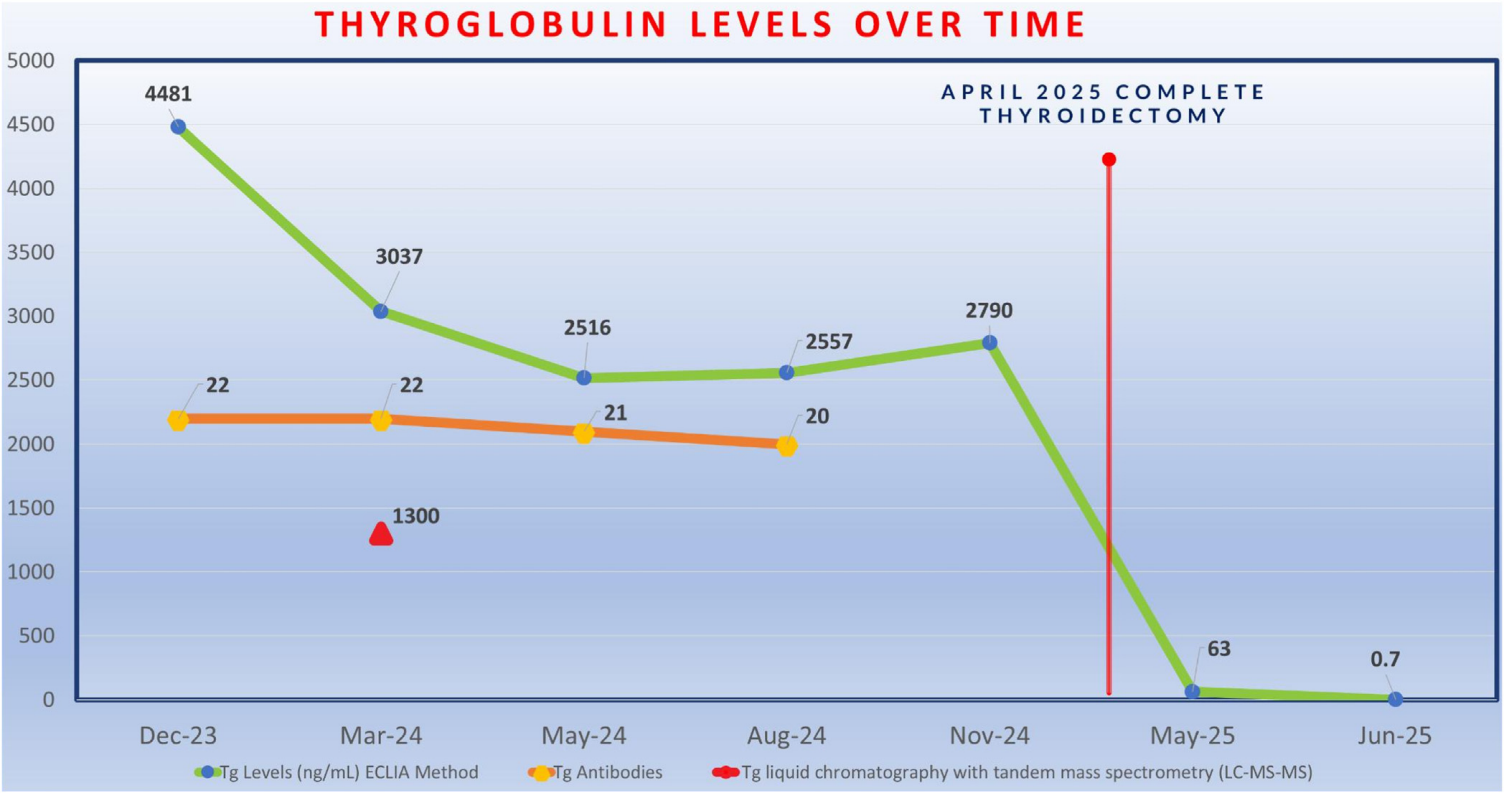
Thyroglobulin trends, measured using various techniques. *ECLIA* = electrochemiluminescence immunoassay.

Given the significantly elevated Tg level measured on electrochemiluminescence immunoassay (Tg-ECLIA) in an otherwise low-risk patient, we considered the possibility of a false laboratory result. A repeated Tg level with Tg-ECLIA technique showed an elevated level of 4481 ng/mL (4481 μg/L), far exceeding the normal range of 3.5-77 ng/mL (3.5-77 μg/L). At this stage, we considered possible assay interference. To investigate further, and considering the known limitation of the ECLIA technique, which can falsely elevate Tg levels due to interference from heterophilic antibodies, we measured Tg using an alternative method: liquid chromatography-tandem mass spectrometry (LC-MS/MS). The Tg level remained elevated at 1300 ng/mL, indicating no heterophilic interference, as it did not normalize and was significantly above the normal range of <33 ng/mL (<33 μg/L). After confirming that the significantly elevated Tg level is accurate by ruling out any false laboratory results or potential heterophilic interference, we carefully assessed the rare possibility of undetected metastatic disease. The patient was closely monitored through repeated biochemical evaluations and structural assessments in a stepwise manner, starting with a thyroid US, which showed a normal appearance of the left thyroid bed and stability of the previously known right thyroid nodule, which measured 2.3 cm. However, her TIRADS classification changed from TIRADS-4 to TIRADS-5 (Fig. 2 *A*–*C*). A subsequent FNA revealed benign cytology for the second time and was classified as Bethesda II. Given the significantly elevated Tg level, we proceeded with further imaging studies, including a chest computed tomography (CT) scan and an 18F-fluorodeoxyglucose positron emission tomography scan. Neither scan showed any evidence of distant metastasis. Therefore, the most likely explanation for the unexpectedly high Tg level is the presence of the benign right thyroid nodule. At this point, the patient opted to undergo a complete thyroidectomy. Following the thyroidectomy, the histopathological evaluation was consistent with a follicular adenoma measuring 3.2 cm. The postoperative serum Tg level fell to 63 ng/mL 4 weeks postoperatively and decreased to 0.7 ng/mL at 8 weeks postoperatively. Given the initial low-risk category and the dramatic reduction of the Tg level after the completion of the thyroidectomy, radioactive iodine (RAI) therapy was not indicated (Fig. 3).

## Discussion

The current report highlights the challenges of interpreting and utilizing serum Tg levels for initial and dynamic risk stratification following TL. There is no defined Tg cutoff point available to assess persistent or recurrent disease after TL, which limits its utility. In the past, TT was recommended for all patients with biopsy-proven DTC measuring 1 cm or larger. This approach typically involved RAI remnant ablation and TSH suppression using levothyroxine. In contrast, TL was primarily reserved for cases of unifocal microcarcinoma smaller than 1 cm.^5^

Currently, a risk-adapted approach is being used, allowing for more individualized treatment plans that determine the extent of initial surgery and whether the patient requires RAI therapy, based on the likelihood of cancer recurrence. More recently, several studies have supported more limited thyroid resection with TL as an equivalent strategy to TT for patients with low-risk DTC in terms of survival with a low percentage of recurrence or the need for completion of thyroidectomy (CT). For instance, Nixon et al reported no differences in local recurrence rates (0% for both TL and TT) and minimal regional recurrence (0% vs 0.8%, *P* = .96) in the TL group compared to the TT. Furthermore, the rate of CT was low, at 6% for immediate cases and 4% for late cases.^6^ As a result of these findings, the 2015 version of the ATA thyroid cancer guidelines recommends TL as an alternative treatment option for low-risk DTC and has expanded the criteria to include patients with tumors measuring up to 4 cm.^3^ Tg is a unique, thyroid-specific protein secreted by the thyroid follicular cells, making it an important parameter to detect both persistent and recurrent disease in DTC after TT and RAI ablation or therapy. Although the 2015 ATA guideline recommends periodic serum Tg measurement along with neck US following TL. However, the prognostic value of post-TL Tg level was always dubitable, as no Tg cutoff value reliably differentiates between normal residual thyroid tissue and recurrent or persistent disease in those patients.^3^^,^^7^^,^^8^

To overcome such a challenge, efforts have been made to establish this Tg point in order to classify the response to therapy for patients who underwent TL. Momesso et al initially suggested that an excellent response can be defined as a nonstimulated, stable Tg level of less than 30 ng/ml, along with negative Tg antibodies and imaging results.^9^ However, interpreting serum Tg levels can be difficult, as studies have shown that Tg levels can rise regardless of whether there is a recurrence of the disease. Ritter et al^8^ conclude that using Tg levels on their own has limited effectiveness for predicting or detecting disease recurrence after TL.

A more recent meta-analysis aimed to evaluate the reliability of Tg measurement after TL in assessing the early response to therapy and predicting the future risk of recurrence. The findings concluded that serum Tg is not reliable for monitoring patients with low-risk DTC treated by lobectomy.^10^ Due to the limitation of Tg monitoring after TL, the Canadian Society of Endocrinology and Metabolism consensus guideline states that monitoring after hemithyroidectomy should rely on neck US.^11^

Tg measured in this case was in line with the previously published 2015 ATA thyroid cancer guidelines as well as the new literature that supports measuring Tg to establish a baseline in case of future concerns.

The first step in addressing this unexpected finding was to rule out any laboratory errors.

We repeated the Tg test to verify the initial result. After confirming the elevated Tg level, we took all necessary measures to eliminate potential interference, particularly from heterophilic antibodies, by retesting the Tg using LC-MS/MS. A difference in Tg levels measured by ECLIA versus LC-MS/MS cannot be interpreted as a true biological reduction in serum Tg concentration. Instead, this discrepancy reflects well-recognized between-method variability, which may exceed 30% even in the absence of TgAb or assay interference. Immunometric assays such as ECLIA are particularly susceptible to heterophilic antibody interference, which can spuriously elevate Tg levels, whereas LC-MS/MS is less affected by such interference but still subject to method-specific bias. For this reason, current guidelines emphasize that longitudinal Tg monitoring should be performed using the same assay platform to ensure consistency and reliability. In this case, LC-MS/MS was employed primarily to exclude heterophilic interference, and subsequent surveillance was continued with the ECLIA platform for trend analysis.^12^

Once we established that the Tg levels were indeed elevated, we focused on ruling out any local or distant recurrence of the disease. This evaluation began with a neck US and a repeated FNA, followed by a chest CT scan and an 18F-fluorodeoxyglucose positron emission tomography scan, all of which confirmed the absence of any disease. Although the initial Tg levels post-TL were much higher than expected based on the volume of the remaining thyroid lobe, based on the histological assessment, her initial risk was considered low risk for recurrence, which was supported by the negative metastasis workup and fall in the Tg level after the completion of the thyroidectomy to the expected range.

In conclusion, we present a challenging finding of significantly elevated Tg levels in low-risk PTC following TL without metastatic disease. In this clinical scenario, the persistent elevation of Tg prior to completion of thyroidectomy should be interpreted as a benign phenomenon related to residual thyroid tissue, not as an indicator of persistent or recurrent cancer. The normalization of Tg after TT and the absence of malignancy on pathology further support this interpretation. This case highlights the limited specificity of Tg measurement for cancer surveillance in patients with an intact thyroid lobe or benign thyroid tissue, underscoring the importance of correlating Tg results with imaging and histopathology.

## Statement of Patient Consent

Signed informed consent was obtained directly from the patient.

## Disclosure

The authors have no conflicts of interest to disclose.
